# Epitope mapping of the monoclonal antibody IP5B11 used for detection of viral haemorrhagic septicaemia virus facilitated by genome sequencing of carpione novirhabdovirus

**DOI:** 10.1186/s13567-023-01166-w

**Published:** 2023-04-17

**Authors:** Takafumi Ito, Tohru Mekata, Niels Jørgen Olesen, Niels Lorenzen

**Affiliations:** 1 Pathology Division, Fisheries Research Agency, Fisheries Technology Institute, 422-1 Nakatsuhamaura, Minami-Ise, Mie 516-0193 Japan; 2 National Institute for Aquatic Resources, Technical University of Denmark, Kemitorvet 202, 2800 Kgs Lyngby, Denmark; 3 Present Address: Faculty of Veterinary Medicine, Okayama University of Science, 1-3 Ikoinooka, Imabari, Ehime Japan

**Keywords:** Epitope mapping, mAb IP5B11, VHSV, *Carpione rhabdovirus*

## Abstract

The monoclonal antibody (mAb) IP5B11, which is used worldwide for the diagnosis of viral haemorrhagic septicaemia (VHS) in fish, reacts with all genotypes of VHS virus (VHSV). The mAb exceptionally also reacts with the *carpione rhabdovirus* (CarRV). Following next generation genome sequencing of CarRV and N protein sequence alignment including five kinds of fish novirhabdoviruses, the epitope recognized by mAb IP5B11 was identified. Dot blot analysis confirmed the epitope of mAb IP5B11 to be associated with the region N219 to N233 of the N protein of VHSV. Phylogenetic analysis identified CarRV as a new member of the fish novirhabdoviruses.

## Introduction, methods, and results

Viral haemorrhagic septicaemia virus (VHSV) belongs to the *Rhabdoviridae* family and the novirhabdovirus genus, as accepted by the International Committee on Taxonomy of Viruses (ICTV) and is known as the causative agent of VHS, a serious disease in farmed and wild fish stocks. VHSV has been found in more than 80 wild and farmed fresh- and seawater fish species in the Northern Hemisphere [1, 2]. Disease outbreaks have significant economic and ecological consequences and new VHSV variants are emerging regularly. New genotypes of VHSV have been reported as IVc in the Atlantic coast of Canada in 2000 [3], IVb in the Great Lakes in North America in 2003 [4], and IVd in the North Atlantic Sea in 2015 [5]. In addition, new hosts for these new genotypes and previously known genotypes have been reported; for example ballan wrasse (*Labris bergylta*) for III [6], mummichog (*Fundulus heteroclitus*) for IVc [3], muskellunge (*Esox masquinongy*) for IVb [4] and lumpfish (*Cyclopterus lumpus*) for IVd [5]. Among the wide variety of VHSV isolates derived from wild marine fish stocks, many display no or low virulence to rainbow trout (*Oncorhynchus mykiss*) [7]. However, variants with high virulence to rainbow trout have evolved from the wild stock reservoirs several times since the first description in 1965 [8–10]. Simple, quick and accurate diagnostic methods are required to survey both wild and farmed fish stocks and prevent spread of the disease. For VHSV, identification is usually done by RT-PCR or by immunoassays based on specifically reacting monoclonal antibodies (mAbs). The mAb IP5B11 [11] which reacts with all VHSV isolates tested so far [12] is used in VHS diagnostics worldwide and is recommended for this purpose in the VHS chapter in the World Organization for Animal Health (WOAH) aquatic manual [2]. However, although it has been reported that mAb IP5B11 recognizes the linear N protein of VHSV in Western blotting [11], details of the epitope recognized by this mAb remain unknown. While mouse mAbs of IgG subclass like IP5B11 may be highly specific at epitope level, particularly linear epitopes may be limited to a relatively short amino acid sequence which implies a certain risk of cross reactions [13]. Therefore, knowledge of the epitope recognized by mAbs used in disease diagnostics is important for evaluating the risk of false positive results.

Back in 1995, it was reported that mAb IP5B11 recognized a rhabdovirus isolated from carpione *Salmo carpio*, a salmonid fish endemic to Lake Garda in Italy [14]. This virus isolate had a protein profile very similar to that of VHSV based on SDS page. Reactivity in Western blotting with mAb IP5B11 appeared to be comparable to that of VHSV, whereas only minor cross reactions were observed for other mono- and polyclonal antibodies [14]. Since mAb IP5B11 binds all tested isolates of VHSV but no other fish viruses, we assumed the mAb IP5B11 epitope could be determined by alignment of the amino acid sequences of the N-proteins of VHSV and related viruses with that of the carpione rhabdovirus. So far, the genetic relationship ofcarpione rhabdovirus with other fish rhabdoviruses has not been studied. Thus, in this study, the full genome of carpione rhabdovirus was analysed by NGS in order to address this issue as well as to clarify its taxonomic position among fish pathogenic rhabdoviruses. Also, following identification of candidate peptides by sequence alignment, the epitope of mAb IP5B11 was determined by dot blot analysis using synthetic oligopeptides.

Apart from the *carpione rhabdovirus* (CarRV) isolate 583 [14] the present study included the VHSV genotype IVa isolate JF00Ehi1 [15] and the *Hirame rhabdovirus* (HIRRV) 8401H isolate [16] as positive and negative controls, respectively, for dot blot analysis. The *Epithelioma papulosum cyprini* (EPC) [17] cell line was used for CarRV propagation. The fathead minnow (FHM) [18] cell line was used for propagation of VHSV JF00Ehi1 and HIRRV. The cell lines were maintained in minimum essential medium supplemented with 10% fetal bovine serum (FBS; Equitech-Bio) and antibiotics (100 IU/mL penicillin and 100 μg/mL streptomycin (FUJIFILM Wako Chemicals). The cultivation of these cell lines was conducted at 25 °C. Each virus isolate was propagated in 75 cm^2^ or 150 cm^2^ flasks at 15 °C. The virus particles were concentrated and sucrose gradient purified from cell culture supernatants as described by Nishizawa et al. [19].

For NGS analysis, EPC cells in a 75 cm^2^ flask were infected with CarRV at a multiplicity of infection (MOI) of 0.01 at 15 °C. Three days after infection, the infected EPC cells were stripped with a cell scraper and pelleted by centrifugation (400 × *g*, 10 min, 4 °C). After removal of the supernatant, total RNA from the cell pellet was extracted with the Direct-zol™ RNA Miniprep (Zymo Research). Extracted RNA solution was sent to Bioengineering Lab. Co., Ltd. for sequencing on a DNBSEQ-G400 (MGI). BLASTN and BLASTX analysis of the resulting contigs were performed using CLC Genomics Workbench (CLC bio) against a database of the National Center for Biotechnology Information (NCBI). The deduced amino acid sequences of the glyco (G)- and the nucleocapsid (N) -proteins of the CarRV were compared by the neighbor joining method with those of other fish pathogenic novirhabdoviruses using MEGA ([20], version 11.0.11).

**Figure 1:**
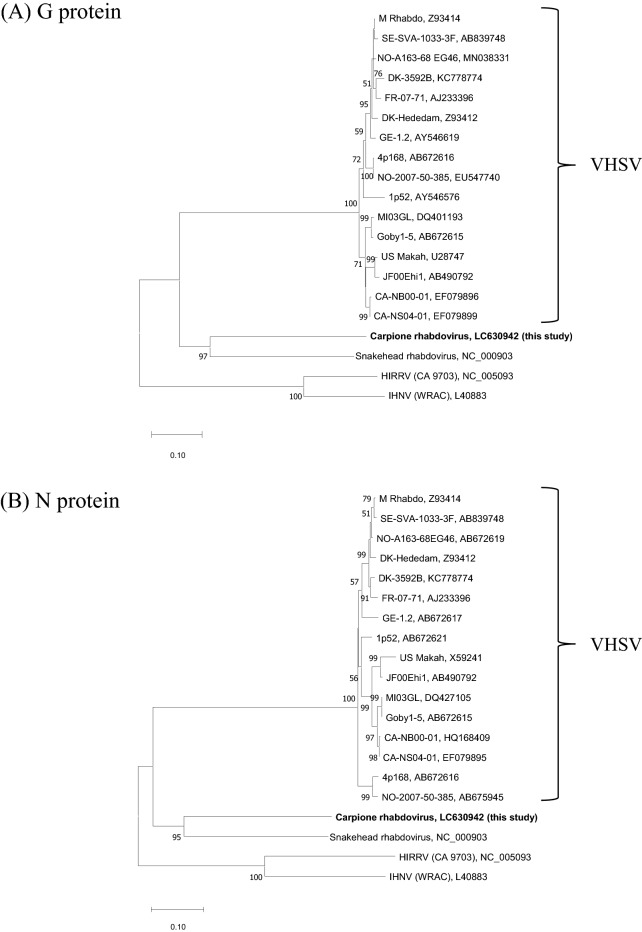
**Phylogenetic tree based on analysis of G and N protein amino acid sequences of piscine Novirhabdovirus by the neighbour joining method**. Sixteen VHSV isolates, representatives of HIRRV, IHNV and SHRV isolates as well as the CarRV isolate 583 were included in the analysis. The accession numbers of the gene sequences used in the analysis are specified next to the names of the virus isolates. Bootstrap values above 50% are shown on the branches.

The alignment analysis included the CarRV, sixteen VHSV isolates representing all known geno- and subtypes, HIRRV [21], Infectious hematopoietic necrosis virus (IHNV) and Snakehead rhabdovirus (SHRV) [22]. The accession numbers of the used gene sequences are specified in Figure 1.

Table 1 shows amino acid identity levels for CarRV G and N proteins respectively, when compared to sixteen VHSV isolates, and SHRV, HIRRV and IHNV representatives. The identity levels between CarRV and VHSV G and N proteins respectively was 45% to 48% for both proteins. The identity of CarRV proteins to SHRV proteins was higher than for VHSV proteins, while the identity of CarRV and IHNV or HIRRV proteins was lower than for VHSV.

**Table 1:** **Identity ratio of amino acid of glycol(G)- and nucleocapsid(N)-proteins among CarRV and sixteen VHSV isolates, SHRV, HIRRV and IHNV isolates**

Virus isolates, genotype	Identity ratio between CarRV 583 (LC630942) (%)^a^	
(Accession number)	G-protein	N-protein
VHSV DK-Hededam, I (Z93412)	45.6	47.8
VHSV DK-3592B, Ia (KC778774)	46.2	47.3
VHSV FR-07–7, Ia (AJ233396)	46.2	46.8
VHSV M rhabdo, Ib (Z93414)	46.2	47.8
VHSV SE-SVA-1033-3F, Ib (AB839748)	46.0	47.8
VHSV NO-A163-68 EG46, Ic (MN038331)	46.2	
(AB672619)		47.5
VHSV GE-1.2, Ie (AY546619)	45.6	
(AB672617)		47.3
VHSV 1p52, II(AY546576)	45.4	
(AB672621)		46.8
VHSV 4p168, III (AB672616)	46.4	46.0
VHSV NO-2007–50-385, III (EU547740)	46.4	
(AB675945)		46.3
VHSV US Makah, IVa (U28747)	47.2	
(X59241)		44.8
VHSV JF00Ehi1, IVa (AB490792)	46.8	
VHSV MI03GL, IVb (DQ401193)	46.8	
(DQ427105)		46.3
VHSV Goby 1–5 (AB672615)	46.6	46.3
VHSV CA-NB00-01, IVc (EF079896)	46.2	
(HQ168409)		46.8
VHSV CA-NS04-01, IVc (EF079899)	46.4	
(EF079895)		46.8
SHRV (NC_000903)	55.0	57.4
HIRRV CA 9703 (NC_005093)	39.9	42.6
IHNV WRAC, M(L40883)	39.3	44.0

^a^Gap-excluded identity.

Dot blot analysis was used for examination of peptide binding specificity. Four synthetic oligopeptides, namely N219–A233; NH2–NGTGMTMIGLFTQAA–COOH (amino acid (aa) positions 219–233 of VHSV isolates), T224–T230; NH2–TMIGLFT–COOH (aa positions 224–230), S251–A256; NH2–SLVESA–COOH (aa positions 251–256), S271–M280; NH2-SIQERYAIMM-COOH (aa positions 271–280) in the N-protein were ordered from Eurofins Genomics. Approximately 5 mg of each oligopeptide was obtained. The N219-A233 was dissolved in 1 mL of distilled water with 20% acetic acid (FUJIFILM Wako Chemicals) and 5% dimethyl sulfoxide (FUJIFILM Wako Chemicals). The T224-T230 was dissolved in 1 mL of distilled water with 20% acetic acid. The S251-A256 and S271-M280 were dissolved in 1 mL of distilled water only. As a positive and negative control for immunostaining, purified VHSV JF00Ehi1 (genotype IVa), carpione rhabdovirus CarRV (583) and HIRRV (8401H) isolates were used. Two microliters of each purified virus and dissolved synthetic oligopeptide were blotted onto a polyvinylidene difluoride membrane (FUJIFILM Wako Chemicals) first pre-wetted in methanol and washed in distilled water. The membrane was incubated with mAb IP5B11 hybridoma cell culture supernatant diluted 1:50 in TBS-T for 1 h at 37 °C after blocking with Blocking One (Nacalai Tesque). After 3 times washing by TBS-T, visualization was performed using horseradish peroxidase-conjugated antiserum to mouse immunoglobulin (Jackson ImmunoResearch) and staining kit (MOSS, #NBTM-500).

The total nucleotide length of CarRV genome was estimated to 11 336 bp. The genome sequence of CarRV has been deposited in National Center for Biotechnology Information (NCBI) GenBank database under the accession no. LC630942. Six open reading frames were identified with greatest BLAST scores to the genes encoding the N protein, polymerase-associated phosphoprotein (P), matrix protein (M), G protein, non-virion protein (Nv), and RNA polymerase (L) of members of the genus *Novirhabdovirus* within the family *Rhabdoviridae*. The N, P, M, G, Nv and L genes of the CarRV encoded proteins of 400, 220, 189, 504, 129, and 1983 aa, respectively.

The presence of the NV-gene implies that the carpione rhabdovirus belongs to the *Novirhabdovirus* genus. The phylogenetic analysis of N and G proteins including the carpione rhabdovirus, VHSV isolates representing all current geno- and subtypes, along with representatives of HIRRV, IHNV and SHRV, further revealed that the CarRV is a unique species, different from VHSV, HIRRV, IHNV and SHRV. In addition, the results suggested that carpione rhabdovirus was most closely related to SHRV (Figure 1).

**Figure 2:**
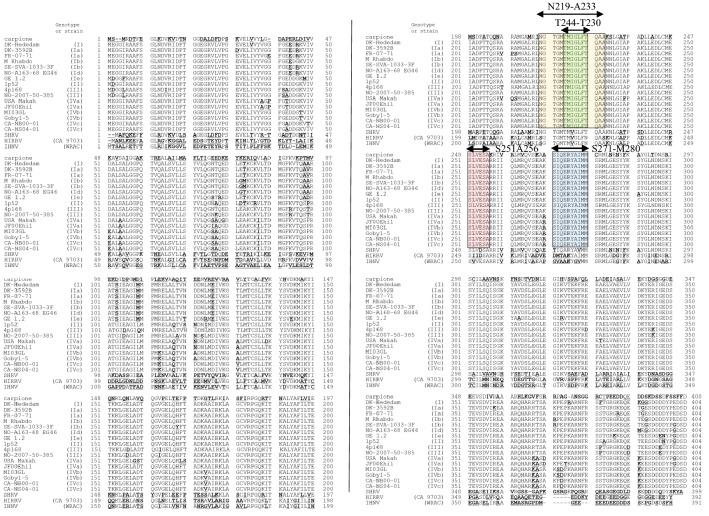
**Amino acid sequence alignment of the N-proteins of CarRV, VHSV, SHRV, HIRRV and IHNV.** Amino acid sequences shaded yellow (aa N219- A233 of the N-protein of VHSV), green (aa T224-T230 of the N-protein of VHSV), red (aa S251-A256 of the N-protein of VHSV) or blue (aa S271-M280 of the N-protein of VHSV) correspond to the synthetic oligopeptides used in epitope mapping of mAb IP5B11. Amino acid substitutions compared to the VHSV consensus sequence are marked in bold and underlined.

Apart from reacting with the CarRV, the N-protein specific mAb IP5B11 is known to react exclusively with VHSV [11, 12]. Since linear epitopes recognized by antibodies may be composed of domains as short as 7 amino acids [23], the N proteins of CarRV, VHSV, IHNV and HIRRV were compared in order to search for 7 + aa long sequences shared exclusively by CarRV and VHSV. Three epitope candidate positions were identified, namely VHSV N219-A233, S251-A256, and S271-M280 (Figure 2).

**Figure 3:**
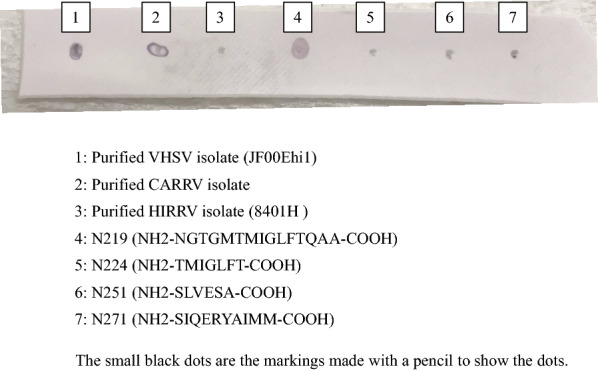
**Epitope mapping of IP5B11 using synthetic oligopeptides in dot-blot analysis.** Purified VHSV isolate (JF00Ehi1) and the CarRV isolate were used as positive controls. Purified HIRRV isolate (8401H) was used as negative control. The purified viruses and synthetic oligopeptides were blotted onto a PVDF membrane. The membrane was incubated with mAb IP5B11 and subsequently immunostained with HRP conjugated secondary antibodies. Dot 1, JF00Ehi1; 2, CarRV; 3, HIRRV; 4, N219-A233 (NH2-NGTGMTMIGLFTQAA-COOH); 5, T224-T230 (NH2-TMIGLFT-COOH); 6, S251-A256 (NH2-SLVESA-COOH); 7, S271-M280 (NH2-SIQERYAIMM-COOH). The size of the stained spots reflected the shape of the sample droplet. All application sites were marked with pencil to specify where samples were applied.

The epitope specificity of mAb IP5B11 was subsequently assessed by dot-blot analysis using the corresponding synthetic oligopeptides. Here mAb IP5B11 was found to bind only peptide N219-A233. In an attempt for further narrow down the epitope, the internal peptide T224-T230 was also included but gave no detectable binding. Reactivity with purified viruses was evident for VHSV JF00Ehi1 and CarRV, but not for HIRRV 8401H (Figure 3).

## Discussion

In this study, full genome sequencing of CarRV was used to taxonomically classify this virus to the *Novirhabdovirus* genus, as well as to determine the epitope of mAb IP5B11 used in immunoassays worldwide for diagnosis of VHSV infections in farmed and wild fish. The results of the genetic analysis of CarRV in this study and serological tests of previous studies [14] fulfil the elements of species demarcation criteria of *Novirhabdovirus* in ICTV, indicating that CarRV should be distinguished from the other viruses. [24]. As the virus name and species name of *Novirhabdovirus* genus is based on the host [24], the appropriate name for this virus is carpione rhabdovirus and the appropriate species name is *Novirhabdovirus carpione*.

The aa sequence of N219-A233 of the N-protein of VHSV and CarRV is NGTGMTM**I**GLFTQAA, while the corresponding aa sequences of SHRV, HIRRV and IHNV are NGTGMTM**V**GLFTQAA, **S**GTGMTM**V**GLFNQA**S** and **S**GTGMTM**V**GLFNQAA, respectively (Figure 2). Therefore, it may be anticipated that Isoleucine I226 contributed significantly to the reactivity of the mAb IP5B11. Since binding of IP5B11 to T224-T230 failed, the epitope probably either depends on a longer peptide or has I226 in a more terminal position.

The IP5B11 epitope has been maintained for all VHSV isolates examined so far without occurring in other salmonid *Novirhabdovirus* species. Hence it may be assumed that the reactivity of mAb IP5B11 with CarRV reflects a closer relationship with VHSV rather than being a result of convergent evolution. Molecular phylogenetic tree analysis of N and G protein sequences accordingly suggested that CarRV is closer to VHSV than to HIRRV and IHNV (Figure 1). Consequently, the IP5B11 epitope region may also be retained in the ancestor of VHSV and CarRV. The CarRV has been isolated from carpione in Lake Garda in Italy [14]. Since Lake Garda is a glacial lake and carpione is a salmonid fish species endemic to Lake Garda [25], the CarRV may have evolved under isolated conditions for thousands of years. Curiously, the phylogenetic analysis also suggested that CarRV is more closely related to SHRV isolated from snakehead fish *Ophicephalus striatus* in Thailand [21] than to VHSV (Figure 1). The lack of IP5B11 binding to SHRV then implies that the latter should have branched from the CarRV later than the branching between VHSV and CarRV. Molecular clock analysis including more novirhabdovirus isolates are required to clarify these interesting issues.

Recently, a new genotype of VHSV termed IVd [5] has been described and it may be assumed that the emergence of other new genotypes will continue. Although the mAb IP5B11 epitope so far has appeared to be highly conserved for VHSV, the dependency of combination of a few amino acids underlines the potential fragility of mAb-based diagnostic assays. Failure to identify a suspect cytopathogenic virus based on lack of recognition by mAb IP5B11 should therefore be supported by other diagnostic means such as RT-PCR [26] or immune-assays including other VHSV specific antibodies [12].

## Data Availability

All data generated or analysed in this study are included in this published article.
